# Supplementary material for: Engraftment, Fate, and Function of HoxB8-Conditional Neutrophil Progenitors in the Unconditioned Murine Host
Source: Front Cell Dev Biol. 2022 Jan 20;10:840894. doi: 10.3389/fcell.2022.840894 (PMC8812959; doi:10.3389/fcell.2022.840894)
Supplement: Supplementary file 1 [file DataSheet1.PDF]

Engraftment, fate, and function of HoxB8-conditional neutrophil progenitors in the unconditioned murine host

Joshua T. Cohen, Michael Danise, Kristina D. Hinman, Brittany M. Neumann, Renita Johnson, Zachary S. Wilson, Anna Chorzalska, Patrycja M. Dubielecka, Craig T. Lefort

Supplementary Information

Supplementary Figure 1

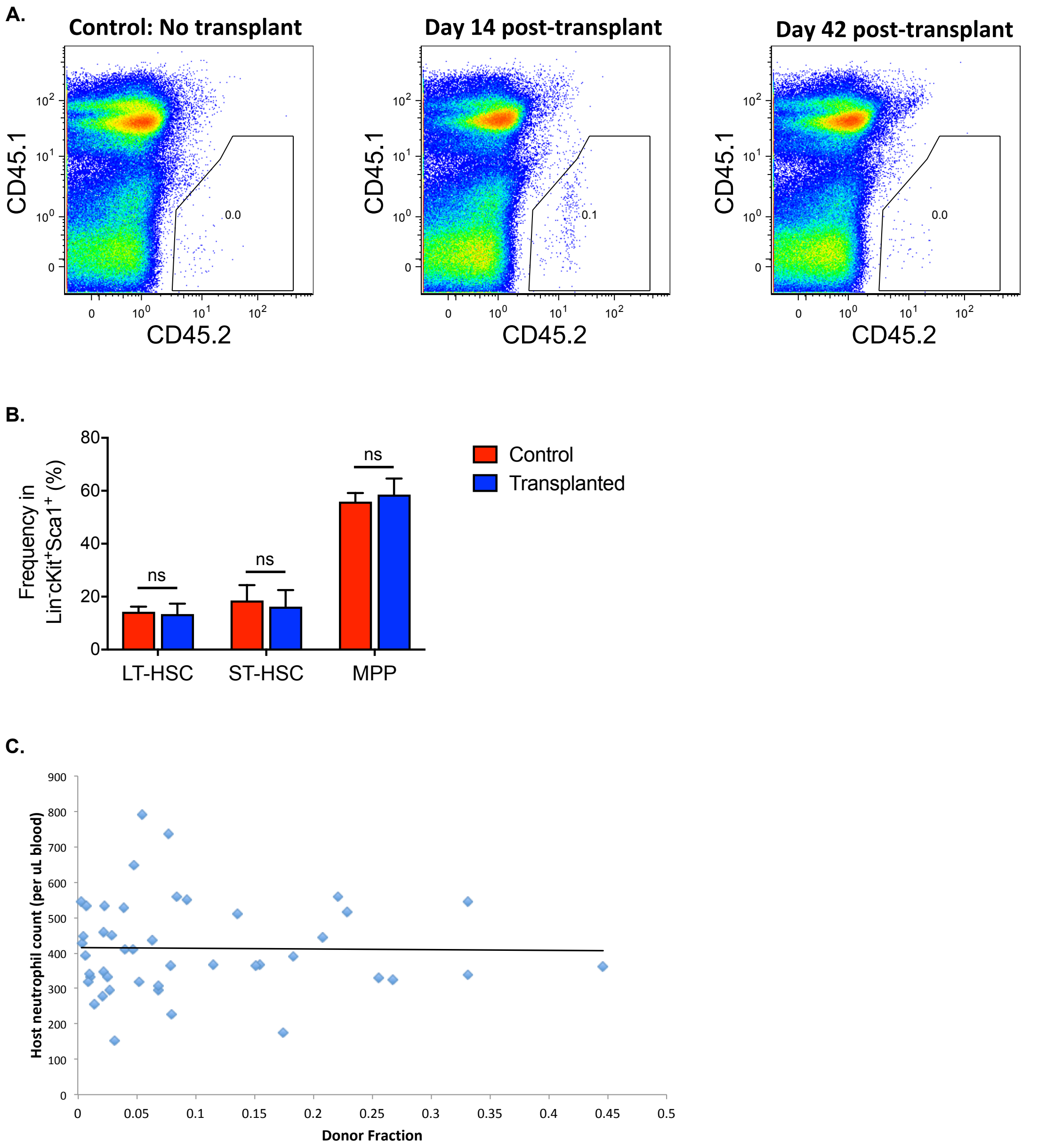

Figure S1. (A) The frequency of donor-derived cells (CD45.2⁺) in whole bone marrow of CD45.1 recipient mice at 2 weeks and 6 weeks after transplantation. (B) Analyses of bone marrow of control mice and those that received HoxB8-conditional progenitor transplantation, as measured at the 6-month time point. Samples were labeled for long-term HSCs (LT-HSC), short-term HSCs (ST-HSC), and multipotent progenitors (MPP) as described in Chorzalska et al. (2018) *Blood*, 132: 2053-2066. (C) Individual measurements of the fraction of donor-derived neutrophils in blood, accrued from serial sampling of CD45.1 mice that received parental or clonal HoxB8-conditional progenitor lines as described in Figure 1D. A linear trendline was projected, indicating a lack of a relationship between donor frequency and absolute neutrophil count in the blood.

Supplementary Figure 2

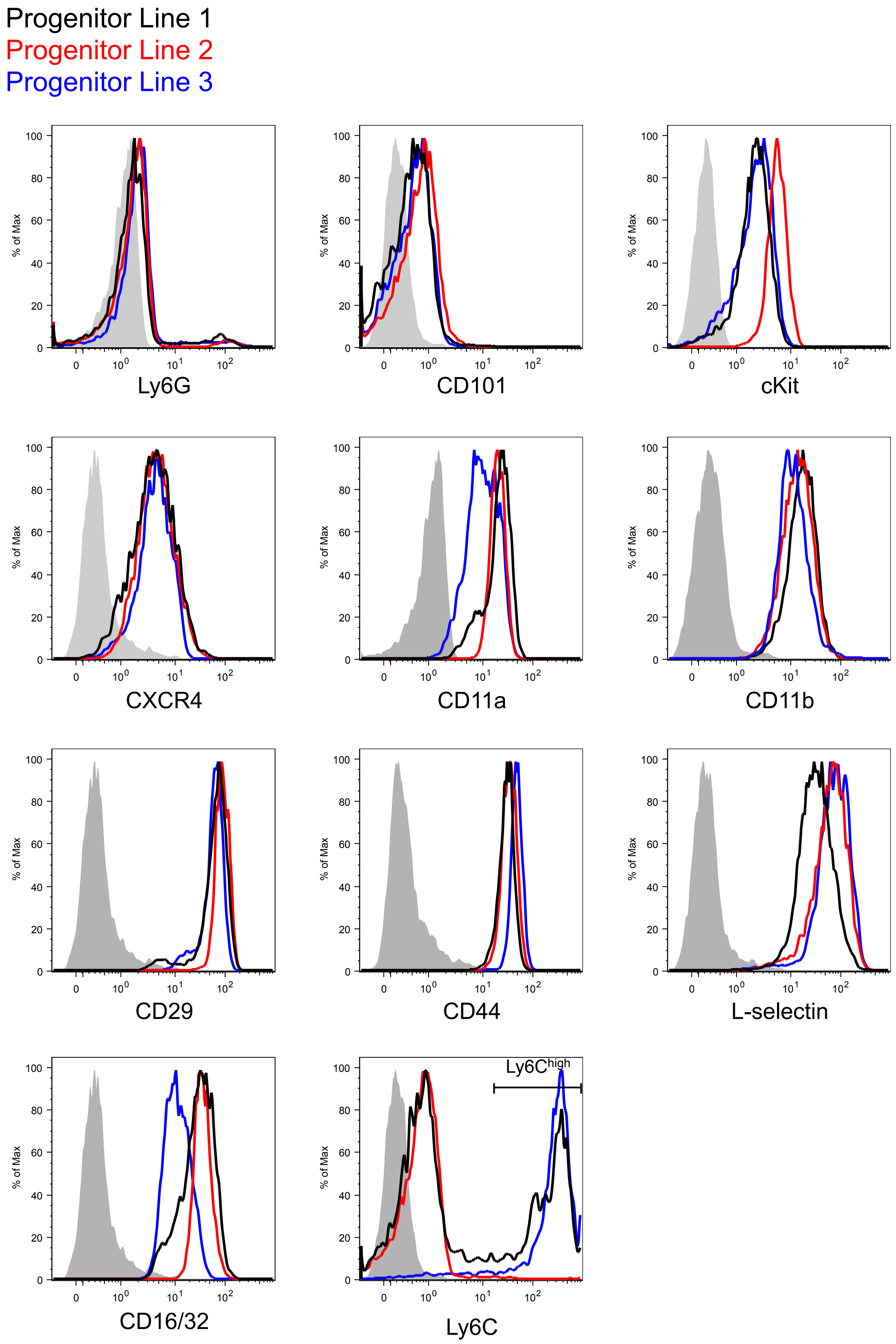

Figure S2. Flow cytometry analyses of the indicated surface receptors and markers on HoxB8-conditional progenitor lines 1, 2, and 3.

Supplementary Figure 3

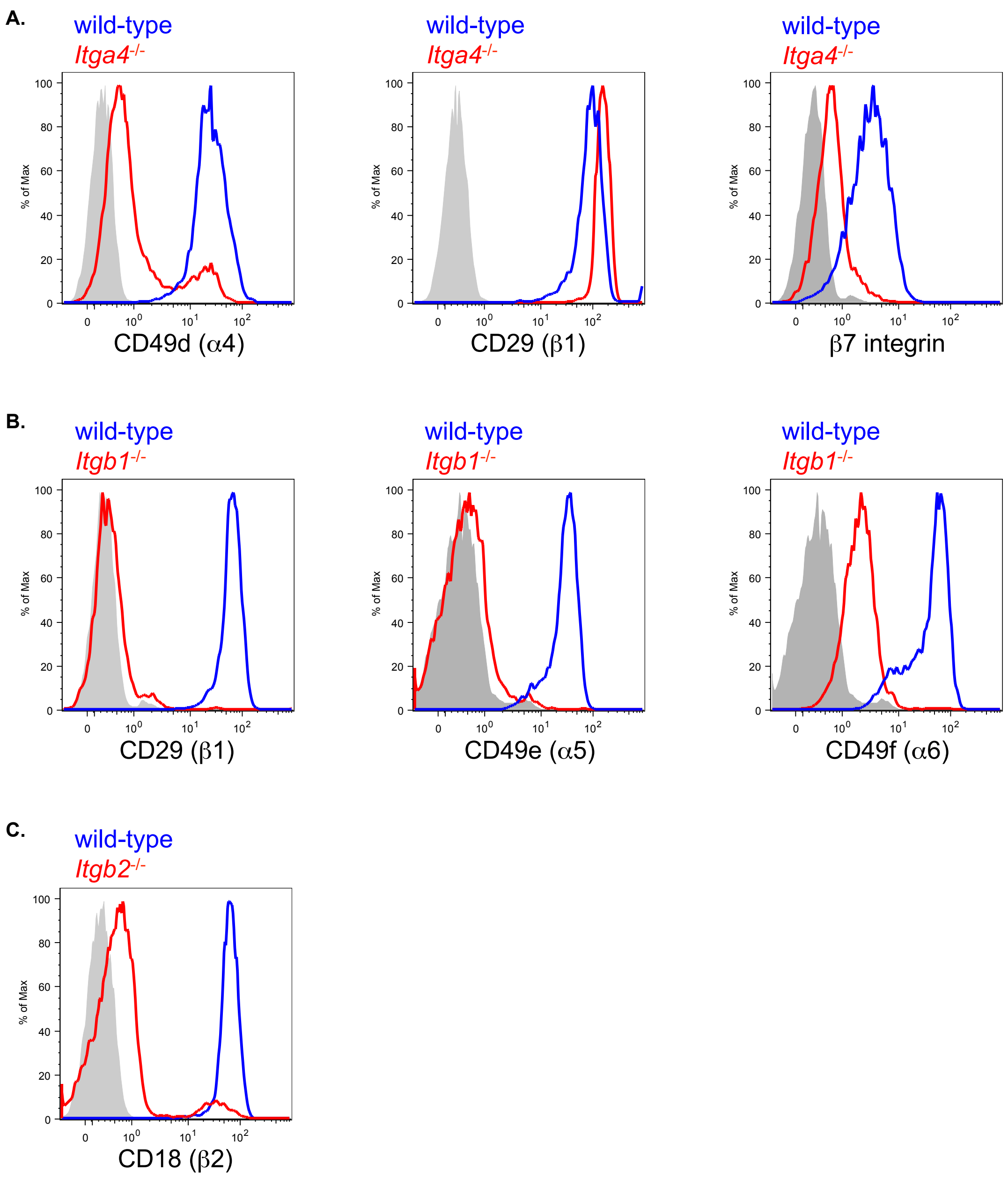

Figure S3. Flow cytometry analyses to determine the expression of the indicated receptors on wild-type and (A) *Itga4*$^{-/-}$, (B) *Itgb1*$^{-/-}$, or (C) *Itgb2*$^{-/-}$ HoxB8-conditional progenitors.

Supplementary Figure 4

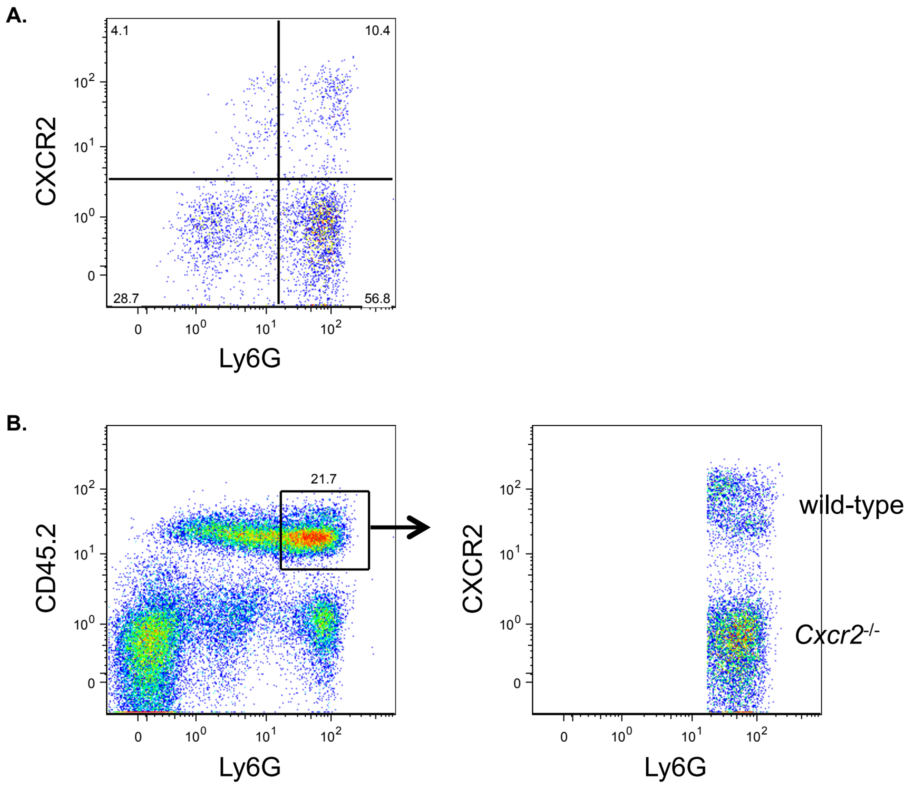

Figure S4. Flow cytometry analysis of donor-derived (CD45.2$^+$) cells in the bone marrow of mice showing (A) that *Cxcr2*$^{-/-}$ HoxB8-conditional progenitors are able to differentiate into Ly6G^{high} neutrophils *in vivo*, and (B) the gating strategy for analyses to determine the fraction of wild-type and CXCR2-deficient donor neutrophils within the donor CD45.2$^+$Ly6G^{high} population of the bone marrow.

Supplementary Figure 5

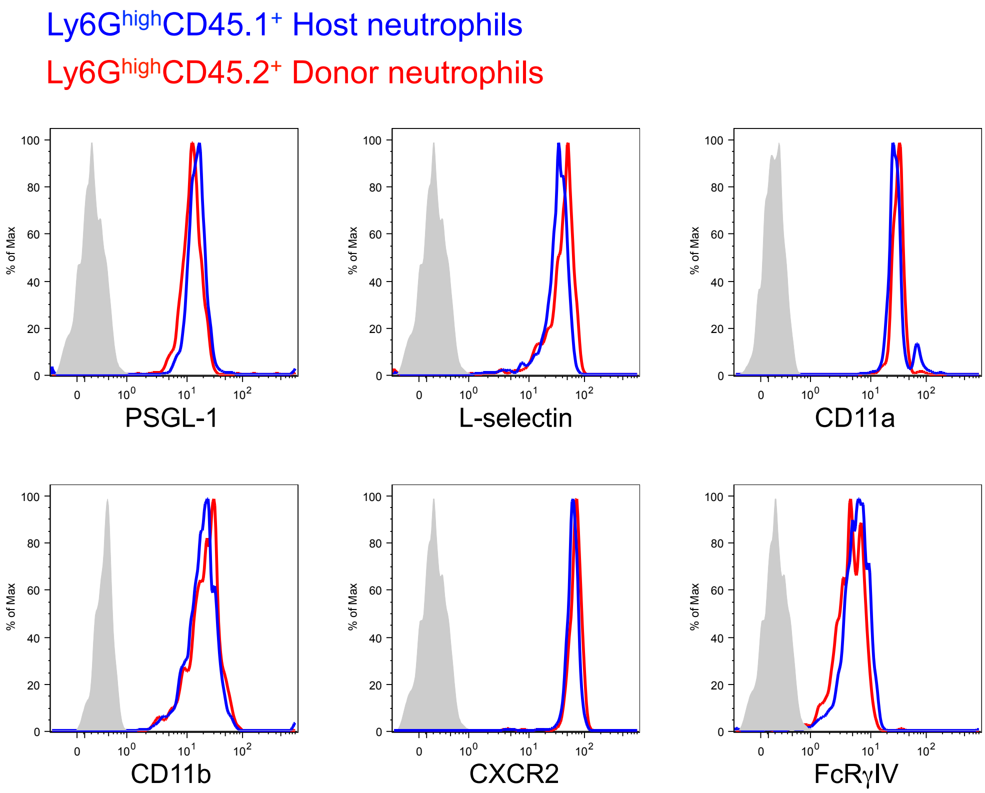

Figure S5. Flow cytometry analyses to determine the expression of the indicated receptors and markers on donor-derived neutrophils ($CD45.2^{+}Ly6G^{high}$) in the blood of CD45.1 recipient mice that were transplanted with wild-type HoxB8-conditional progenitors, and their comparison to host-derived neutrophils ($CD45.1^{+}Ly6G^{high}$) in the same blood sample.

Supplementary Figure 6

Time: 0 min

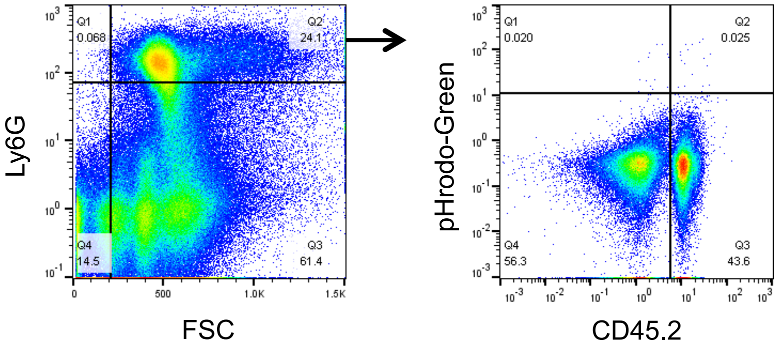

Time: 60 min

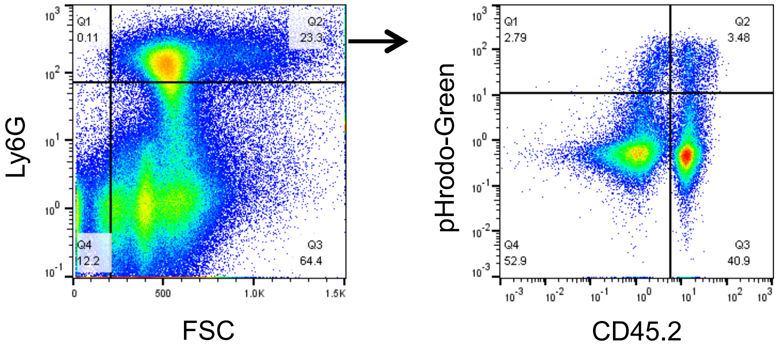

Figure S6. Flow cytometry analyses of phagocytosis assays performed with bone marrow samples. These data indicate the gating strategy to analyze and determine the fraction of donor- and host-derived neutrophils that internalized pHrodo-Green *S. aureus*, at 0 and 60 min time points.
